# Diagnostic Accuracy of Predictive Models in Prostate Cancer: A Systematic Review and Meta-Analysis

**DOI:** 10.1155/2022/1742789

**Published:** 2022-06-08

**Authors:** Mohammad Saatchi, Fatemeh Khatami, Rahil Mashhadi, Akram Mirzaei, Leila Zareian, Zeinab Ahadi, Seyed Mohammad Kazem Aghamir

**Affiliations:** ^1^Urology Research Center, Tehran University of Medical Sciences, Tehran, Iran; ^2^Department of Epidemiology and Biostatistics, School of Public Health, Tehran University of Medical Sciences, Tehran, Iran

## Abstract

**Aim:**

Accurate diagnosis of prostate cancer (PCa) has a fundamental role in clinical and patient care. Recent advances in diagnostic testing and marker lead to standardized interpretation and increased prescription by clinicians to improve the detection of clinically significant PCa and select patients who strictly require targeted biopsies.

**Methods:**

In this study, we present a systematic review of the overall diagnostic accuracy of each testing panel regarding the panel details. In this meta-analysis, using a structured search, Web of Science and PubMed databases were searched up to 23 September 2019 with no restrictions and filters. The study's outcome was the AUC and 95% confidence interval of prediction models. This index was reported as an overall and based on the WHO region and models with/without MRI.

**Results:**

The thirteen final articles included 25,691 people. The overall AUC and 95% CI in thirteen studies were 0.78 and 95% CI: 0.73–0.82. The weighted average AUC in the countries of the Americas region was 0.73 (95% CI: 0.70–0.75), and in European countries, it was 0.80 (95% CI: 0.72–0.88). In four studies with MRI, the average weighted AUC was 0.88 (95% CI: 0.86–0.90), while in other articles where MRI was not a parameter in the diagnostic model, the mean AUC was 0.73 (95% CI: 0.70–0.76).

**Conclusions:**

The present study's findings showed that MRI significantly improved the detection accuracy of prostate cancer and had the highest discrimination to distinguish candidates for biopsy.

## 1. Introduction

Prostate cancer (PCa) is the second most frequent cancer in men worldwide, and its incidence and mortality correlate with increasing age [1]. The accurate PCa diagnosis is a problematic issue because it is essential to identify which PCa are destined to progress and which would benefit from early radical treatment [2]. PCa has traditionally been diagnosed by digital rectal examination (DRE) and prostate-specific antigen (PSA) blood test, followed by transrectal ultrasound (TRUS) guided biopsy [3]. Their limited specificity and an elevated rate of overdiagnosis are the main problems associated with PCa testing. Benign prostatic hyperplasia (BPH) has similar symptoms to PCa, and most PCa patients are diagnosed as asymptomatic patients with normal DRE and elevated PSA [4].

So, new PCa biomarkers have been proposed to improve the accuracy of PSA in the management of early PCa [5–8]. The diagnostic panels include PSA isoforms, PSA density and velocity, age-adjusted PSA, free PSA to total PSA ratio (fPSA/tPSA), PSA density (PSAd), PSA doubling time (PSADT), Prostate health index (Phi), 4K score (include kallikrein-related peptidase 2/hK2, intact PSA, fPSA, and tPSA), advanced MRI (mpMRI and bpMRI), PCA3 mRNA, PSA glycoforms, TMPRSS2:ERG fusion gene, microRNAs, circulating tumor cells (CTCs), and androgen receptor variants [5, 9–12]. AUC is an effective way to summarize the overall diagnostic accuracy of each testing panel.

No comprehensive study represents the most accurate ones, and the heterogeneity of all clinical trials is too high in both the panel components and AUC. This systematic review summarizes all PCa diagnostic panels and compares their AUC to find the most accurate ones.

## 2. Methods

This systematic review and meta-analysis were designed according to the latest version of the PRISMA checklist, and it was registered on Prospero with registration number: CRD 149417. The summary major was AUC with a 95% confidence interval (CI).

### 2.1. Search Strategy

We searched Scopus, Web of Science, and PubMed databases on 23 September 2019. The search query was as follows: “Prostate Neoplasms” OR “Prostatic Neoplasm” OR “Prostate Cancer” OR “Prostatic Cancer” AND “ risk score” OR “prognostic score” OR “prognostic model” OR “prognostic panel” OR “prognostic score model”. Duplicate studies were removed prior to download. After that, we included articles with these inclusion criteria: articles that provided a model/panel for prostate cancer prediction. Exclusion criteria included the following: (1) articles that investigate genetic factors, (2) articles that did not report AUC (with 95% confidence interval) for their model, and (3) articles studying the treatment, recurrence, or metastasis of prostate cancer.

### 2.2. Data Collection

Three reviewers, RM, AM, and LZ were independently involved in the title and abstract and read and determined the eligibility of the studies. All three authors, RM, AM, and LZ, independently extracted all relevant data, including the year of publication, first author, country, sample size, mean/median or range of age, AUC (95% CI), and model contents. The disagreement was resolved by discussion, and when necessary, two reviewers (SMKA and FKh) assisted in adjudicating a final decision.

### 2.3. Methodological Quality Assessment

The Newcastle–Ottawa Scale (NOS) assessment tool was used to evaluate the quality of the articles by three authors [1]. The scoring was based on the assignment of stars from 1 to 9. According to the NOS score, the selected studies were divided into high quality (≥6) and low quality (<6).

### 2.4. Statistical Analysis

The chi-square test at a significant level of 5% was used for the qualitative assessment of heterogeneity across studies. Based on the Higgins categorization, an I-square of more than 75% was considered heterogeneity. The index of interest in this study was AUC which was calculated as the proportion using the ROC curve method with 95% confidence intervals. The weights for the weighted average AUC calculation were calculated in accordance with the methods described by Zhou et al. [13]. Data analysis was performed using the Stata version 11 (StataCorp, College Station, TX, USA) statistical software. Also, the random effect model at a confidence level of 95% was used in the data analysis.

## 3. Results

**Figure 1:**
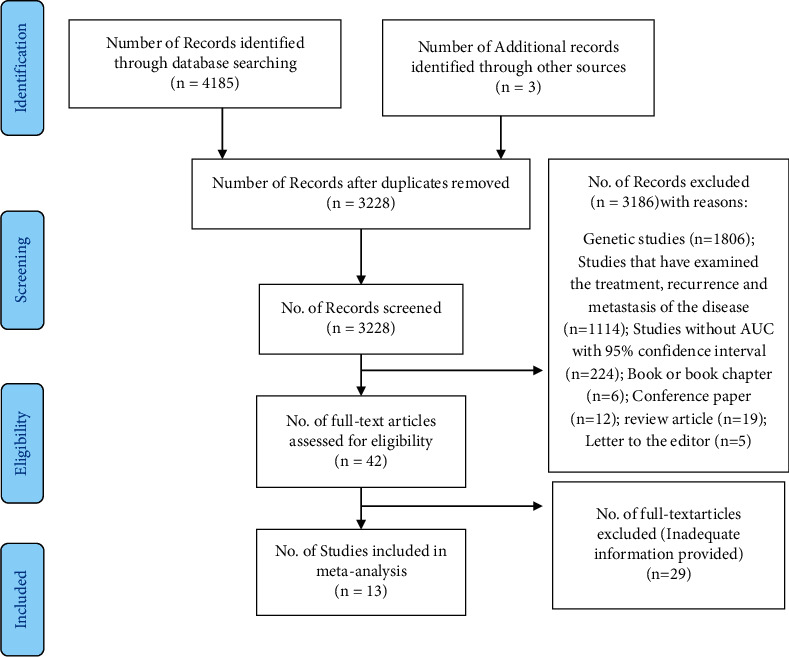
Flow of information through different steps of the systematic review and meta-analysis.

In this systematic review, 4188 articles were identified, of which 4185 articles were extracted from the search of electronic databases, and three articles were extracted from the search of the list of selected articles and other sources. After deleting duplicate articles, the title and abstract of 3228 articles were screened, and according to the exclusion criteria, 3186 articles were removed. Finally, 13 articles were used in the final analysis (Figure 1).

The thirteen final articles included 25,691 people. The characteristics of the studies include the names of the authors, the country, the WHO region, sample size, mean or median age, AUC and 95% confidence interval, model parameters, quality assessment score, and model name. Based on the findings of our study, the highest AUC was observed in the study of Boesen et al. [14] (0.89 (0.87–0.92)) and Dwivedi et al. [15] (0.89 (0.83–0.95)). In both studies, MRI played an important role in increasing AUC. In the study of Roobol et al. [16], the lowest AUC was observed in the GOTEBORG-R2–6 cohort and PSA DRE-model, which included only PSA, DRE, and Prior biopsy (Table 1).

**Figure 2:**
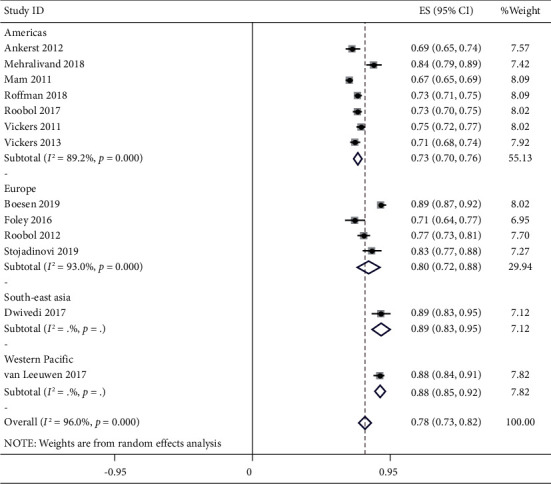
Forest plot of AUC (95% CI) of predictive models in the different regions.

In the final analysis, most articles are from European and American regions. As shown in Figure 2, the weighted average AUC in the countries of the American region was 0.73 (95% CI: 0.70–0.75), and in European countries, it was 0.80 (95% CI: 0.72–0.88). A study from Southeast Asia and a study from the Asia-Pacific region were also in the final analysis. The overall AUC and 95% CI in thirteen studies was 0.78 (95% CI: 0.73–0.82). Data from the previously published meta-analysis indicated that PI-RADS are superior in diagnosing PCa with high sensitivity, specificity, and AUC than PHI and PCA3 [22].

**Figure 3:**
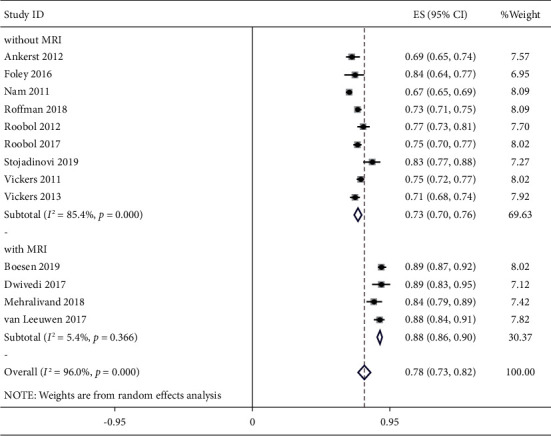
Forest plot of AUC (95% CI) of predictive models according to with/without MRI.

Figure 3 shows the AUC of studies based on the presence or absence of MRI in the final model. In four studies with MRI, the average weighted of AUCs was 0.88 (95% CI: 0.86–0.90), while in other articles where MRI was not a parameter in the diagnostic model, the mean AUCs were 0.73 (95% CI: 0.70–0.76).

**Figure 4:**
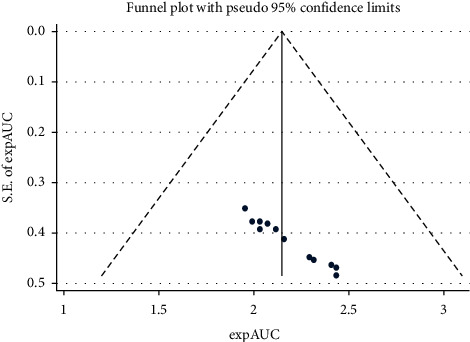
Funnel plot to assess the publication bias.

Figure 4 shows the funnel plot to investigate publication bias. The Begg (*P* value = 0.428) and Egger (*P* value = 0.780) tests showed no significant publication bias in our study.

## 4. Discussion

The present study assessed the predictive models for PCa detection to find the models that had the highest discrimination in distinguishing candidates for biopsy. In the current study, the highest AUCs were observed for two models; one of them is based on age, PSA density, DRE, and bpMRI (AUC: 0.89, 95% CI: 0.87–0.92) [23], and the second one developed with PSA, MRSI, and DW-MRI (mpMRI) (AUC: 0.89, 95% CI: 0.83–0.95) [14]. The present study's findings showed that the best predictive models for PC detection were based on the combination of clinical parameters and bpMRI or mpMRI. By adding the MRI to clinical parameters, the predictive accuracy improved significantly. Also, the AUCs of most models based on only clinical variables were lower than the AUCs of models with the incorporation of imaging [16–21, 24, 25].

Previous documents assessing the efficacy of prostate cancer detection have highlighted the need to decrease insignificant prostate cancer's overdiagnosis [26–28]. Hence, a novel diagnostic panel is required to decrease the number of unneeded biopsies and recognition of insignificant prostate cancer. So, recently numerous nomograms and predictive models with various parameters, varying degrees of accuracy, generalizability, and validation were developed to improve the accuracy of PC diagnosis. Recently, the evidence showed that when mpMRI or bpMRI is added to the standard clinical factors, the predictive accuracy enhances [14, 23, 29, 30]. A meta-analysis showed that bpMRI offers similar test accuracy to mpMRI in identifying prostate cancer, but heterogeneity does not allow definitive recommendations to be made [31]. Boesen et al. [14] showed that by adding bpMRI to clinical parameters (age, PSAd, ctDRE), the AUC of the model improved significantly from 0.85 to 0.89 for predicting PC and achieved the highest discrimination power. Also, they showed that the AUC of the model based on the only bpMRI was 0.84 and demonstrated that the MRI-derived score as a PC detection is the most powerful single predictor. In line with this, Dwivedi et al. [23] found that the model's accuracy is higher with mpMRI than without (0.89 vs. 0.66). van Leeuwen et al. showed that the addition of mpMRI to commonly used clinical elements enhanced the predictive accuracy by 9% [29]. As a result, MRI, along with clinical parameters, can be utilized to decrease the number of unnecessary biopsies. Otherwise, MRI can ensure information about cancer location, staging, and the volume for target biopsies. At present, both the American and European associations of urology (AUA and EAU) recommend using mpMRI as a useful diagnostic tool before repeat biopsy and for men enrolled in active surveillance [32, 33]. A recent systematic review reported clinically significant disease detection rates, the sensitivity, and the negative predictive value (NPV) of mpMRI ranged from 44 to 87%, 58–97%, and 63% to 98%, respectively. In 2022, Futterer et al. had shown that mpMRI could be applied to rule out significant disease because of its extraordinary NPV [34]. The use of radiomics and kallikreins failed to outperform PI-RADSv2.1/IMPROD bpMRI Likert, and their combination did not lead to further performance gains. The high expenses of mpMRI are debating using the mpMRI to detect prostate cancer. Despite the high cost of mpMRI at first look, it is generally considered a cost-effective method in PC diagnosis because it reduces unnecessary biopsies costs, prevents unnecessary therapies, and increases the quality of life in the long term [35, 36]. The prostate-specific membrane antigen (PSMA) PET/CT and mpMRI have comparable diagnostic accuracy in the discovery and intraprostatic localization of prostate cancer foci whereas mpMRI makes better in the assessment of extracapsular extension (ECE) and seminal vesicle invasion (SVI) [37]. However, the advantage of systematic biopsy (SBx) added to combined MRI/ultrasound fusion targeted biopsy (TBx) is mainly limited to smaller PI-RADS score 3–4 lesions [38].

In the current study, the highest AUC of the model developed based on only clinical variables was 0.83, and this model was developed with age, PSAd, DRE, prostate volume, and PSA [39]. The determining PSAd requires an accurate assessment of prostate volume, and in this model, prostate volume was estimated using transrectal ultrasound (TRUS) in the three dimensions. The accuracy assessment of prostate volume using DRE has been found to be insufficient [40]. Furthermore, TRUS has been the first choice among imaging modalities for a long time [41]. However, low intraoperator reproducibility and the poor interoperator agreement could affect the accuracy of TRUS [42].

Additionally, prostate volume might be underestimated using RTUS, particularly in patients with prostatic hyperplasia [43–45]. Recently, MRI has played a critical role in detecting PC and is considered the greatest accurate and reliable imaging for prostate volume estimation [46, 47]. For example, Boesen et al. achieved the AUC of 0.85 and excellent discrimination by the combination of only three clinical parameters, including age, PSAd, and ctDRE [14], and in this study, the prostate volume was evaluated using bpMRI and indicated that MRI has excellent accuracy in estimating prostate volume.

There were significant differences in the AUCs of predictive models between South East Asia and the Western Pacific with the Americas region (0.89 vs. 0.73, 0.88 vs. 0.73, respectively). Europe, the Western Pacific, and Southeast Asia regions had similar AUCs. The highest AUCs were observed in studies where models were developed based on MRI and clinical markers in all four regions. The variance of AUCs can probably be explained by inherent differences in study design, calculation methods of AUCs, various parameters of models, and validation methods of AUCs.

The overall AUC of models that were based on the combination of MRI and clinical parameters was 0.88, and the AUC of models that were developed with only clinical variables was 0.73. There was a significant difference between the accuracy of models with and without MRI. The findings indicated that the addition of MRI could improve the accuracy of predictive models by 15%.

*Limitation*. There was wide heterogeneity across the studies in terms of study design, calculation methods of AUCs, various parameters of models, and validation methods of AUCs. Therefore, these factors might assemble highly heterogeneous studies, and the finding of our study showed the high heterogeneity and this subject is inevitable. Also, another limitation of the study was the search date. We searched databases until September 2019, and the studies published after this date were not included in the review and meta-analysis.

## 5. Conclusion

The present study confirmed that mpMRI and bpMRI were the strong predictive markers to improve the detection accuracy of models and could decrease the rate of unnecessary biopsies and decrease the overdetection of insignificant prostate cancer.

## Figures and Tables

**Table 1:** Baseline characteristics for studies included in meta-analysis.

ID	Author Name	Country	WHO region	Sample size	Mean age/range/median	AUC	Model content	Score	Model name
1	Ankerst et al. [17]	USA	Americas	575	63.4	0.69 (0.65–0.74)	Total PSA Family history DRE Free PSA	7	Prostate Cancer Prevention Trial Risk Calculator (PCPTRC) model
						0.64 (0.65–0.74)	Total PSA Family history DRE Free PSA [-2] Pro PSA		

2	Boesen et al. [14]	Denmark	Europe	876	65	0.89 (0.87–0.92)	Age PSA density cTDRE bpMRI	8	Advanced imaging model
						0.78 (0.75–0.82)	PSA cTDRE		Baseline model
						0.84 (0.81–0.86)	bpMRI		Imaging model
						0.85 (0.83–0.88)	Age PSA density cTDRE		Advanced model

3	Dwivedi et al. [15]	India	Southeast Asia	137	65	0.66 (NA)	Age ADC PSA Metabolic ratio	9	Original
						0.78 (NA)	Age ADC PSA Metabolic ratio DW-MRI		Original
						0.83 (NA)	Age ADC PSA Metabolic ratio MRSI		Original
						0.89 (0.83–0.95)	Age ADC PSA Metabolic ratio mpMRI (MRSI + DW-MRI)		Developed model

4	Foley et al. [18]	Ireland	Europe	250	63.7	0.71 (0.64–0.77)	Age at biopsy Abnormality on DRE Family history Previous negative biopsy Total PSA Free PSA p2PSA	7	Predicting PHI
						0.62 (0.55–0.69)	Age at biopsy Abnormality on DRE Family history Previous negative biopsy PSA		Predicting PSA
5	Nam et al. [19]	Canada	Americas	2130	Median age 63	0.67 (0.65–0.69)	Age Family history Ethnicity Urinary voiding Symptom score DRE PSA free: total PSA ratio	8	Sunnybrook nomogram-based prostate cancer risk calculator (SRC)
						0.61 (0.59–0.64)	Age Family history Ethnicity DRE PSA		Prostate Cancer Prevention Trial (PCPT)-based risk calculator (PRC)

6	Roffman et al. [20]	USA	Americas	1672	67	0.73 (0.71–0.75)	Age BMI Diabetes status Smoking status Emphysema Asthma Race Ethnicity Hypertension Heart disease Exercise habits History of stroke	9	Multi parameterized artificial neural network (ANN)

7	Roobol et al. [21]	Netherland	Europe	3580	68	Low-risk PCa 0.70 (0.68–0.72)	Age PSA (class via DRE) Abnormal DRE Prostate volume	9	DRE-model
						Low-risk PCa 0.73 (0.70–0.75)	PSA Age Abnormal DRE Prostate volume Abnormal TRUS		TRUS model
8	Roobol et al. [16]	Netherlands	Europe	740	Median age 61	0.77 (0.73–0.81)	PSA DRE Prostate volume Prior biopsy	8	GOTEBORG-R1 cohort DRE vol-RC model
				740	Median age 61	0.71 (0.67–0.76)	PSA DRE Prior biopsy		GOTEBORG-R1 cohort PSA DRE-model
				1241	Median age 63	0.60 (0.57–0.64)	PSA DRE Prostate volume Prior Biopsy		GOTEBORG-R2–6 cohort DRE vol-RC model
				1241	Median age 63	0.56 (0.52–0.60)	PSA DRE Prior biopsy		GOTEBORG-R2–6 cohort PSA DRE-model
				2895	Median age 66	0.74 (0.72–0.79)	PSA DRE Prostate volume Prior biopsy Family history		ROTTERDAM-R1 cohort DRE vol-RC model
				1494	Median age 67	0.65 (0.62–0.69)	PSA DRE Prostate volume Prior biopsy Family history		ROTTERDAM-R2-3 cohort DRE vol-RC model
				1494	Median age 67	0.60 (0.57–0.63)	PSA DRE Prior biopsy Family history		ROTTERDAM-R2-3 cohort PSA DRE-model
				2631	Median age 64	0.66 (0.64–0.68)	PSA DRE Prostate volume Biopsy Gleason grade Family history African origin Prior biopsy		CCF cohort DRE vol-RC model
				2631	Median age 64	0.62 (0.60–0.64)	PSA DRE Biopsy Gleason grade Family history African origin Prior biopsy		CCF cohort PSA DRE-model
				4199	Median age 63	0.72 (0.70–0.73)	PSA DRE Prostate volume Prior biopsy		Tyrol cohort DRE vol-RC model
				4199	Median age 63	0.67 (0.65–0.69)	PSA DRE Prior biopsy		Tyrol cohort PSA DRE-model

Abbreviations. PSA: prostate-specific antigen, DRE: digital rectal examination, PCPTRC: prostate cancer prevention trial risk calculator, PRC: prostate cancer prevention trial (PCPT)-based risk calculator, ANN: artificial neural network, TRUS: transrectal ultrasound, DW-MRI: diffusion-weighted magnetic resonance imaging, BMI: body mass index, SRC: Sunnybrook nomogram–based prostate cancer risk calculator, MRSI: magnetic resonance spectroscopic imaging, ADC: apparent diffusion coefficients, and PHI: prostate health index.

## Data Availability

The data will be provided by the corresponding author on request.
